# Contribution of hepatitis B virus X protein-induced aberrant microRNA expression to hepatocellular carcinoma pathogenesis

**DOI:** 10.3906/biy-1807-196

**Published:** 2019-04-05

**Authors:** Zhiyuan WEI, Xiaohe SHEN, Bing NI, Gaoxing LUO, Yi TIAN, Yi SUN

**Affiliations:** 1 Department of Microbiology and Immunology, Shanxi Medical University , Taiyuan, Shanxi , P.R. China; 2 Department of Pathophysiology and High Altitude Pathology, Army Medical University (Third Military Medical University) , Chongqing , P.R. China; 3 Institute of Immunology, PLA, Army Medical University (Third Military Medical University) , Chongqing , P.R. China; 4 Southwest Hospital, Army Medical University (Third Military Medical University) , Chongqing , P. R. China

**Keywords:** Hepatocarcinoma, HBX, miRNA, internetwork

## Abstract

The hepatitis B virus-encoded X (HBX) protein plays important roles in Hepatocellular carcinoma (HCC). Previous studies have demonstrated that HBX can induce alterations in the expression of numerous microRNAs (miRNAs) involved in the carcinogenesis of various tumors. However, the global profile of liver miRNA changes induced by HBX has not been characterized. In this study, we conducted a miRNA microarray analysis to investigate the influence of HBX on the expression of total miRNAs in liver in relation to HCC. Comparative analysis of the data from human normal liver cells (L02) and human HCC cells (HepG2), with or without HBX, identified 19 differentially expressed miRNAs, including 5 with known association to HBX. Target gene prediction for the aberrantly expressed miRNAs identified a total of 304 potential target genes, involved in sundry pathways. Finally, pathway analysis of the HBXinduced miRNAs pathway showed that 5 of the total miRNAs formed an internetwork, suggesting that HBX might exert its pathological effects on hepatic cells through functional synergy with miRNAs that regulated common pathways in liver cells. Therefore, this work provides new insights into the mechanisms of HCC as well as potential diagnostic markers or therapeutic targets for use in clinical management of HCC.

## 1. Introduction

Hepatocellular carcinoma (HCC) remains one of the most lethal malignant cancers worldwide, ranking third among all the cancers for annual cancer mortality. Chronic infection with hepatitis B virus (HBV) is a major etiological risk for the development and progression of HCC (Kew, 2010) . The mechanisms underlying HBVinduced malignant transformation remain a topic of intense research, and recent studies have revealed that the hepatitis B virus-encoded X (HBX) protein, which is essential for virus replication in vivo, plays an important role in hepatocarcinogenesis (Chen et al., 1993) .

HBX-induced HCC involves disruption of the signaling pathways that control normal physiological functions in the host cells (Tian et al., 2013) . For instance, the HBX protein can serve as a substrate of protein kinase B (Akt kinase) (Srisuttee et al., 2012) , the subsequent dysregulation of which affects pathways that mediate cell survival and oncogenic transformation (Vivanco and Sawyers, 2002) . Ectopic expression of HBX in human normal liver cells (L02, stably transfected with HBX) leads to significantly increased activity of the multifunctional Notch1 signaling pathway and marked inhibition of apoptosis via the caspase 9-caspase 3 signaling pathway (Sun et al., 2014) . Furthermore, HBX can interrupt the DNA repair process through its regulation of the transactivating function of p53 (Lee et al., 2005) .

HBX can also induce aberrant expression of microRNAs (miRNAs), and this infection-related process has been shown to contribute to the pathogenesis of HCC (Esteller, 2011; Xu et al., 2013; Wu et al., 2014) . miRNAs can function on either oncogenes or tumor suppressor genes to mediate tumorigenesis (Wei et al., 2015) . The presence of HBX has been shown to be significantly associated with alterations in the host miRNA profile (Trang et al., 2008) . For example, it suppresses the expression of miRNA-148a, which results in the activation of AKT and of the extracellular signal-regulated kinase signaling pathway, ultimately leading to the activation of rapamycin and the subsequent promotion of cancer cell proliferation and metastasis, as shown in a mouse model (Xu et al., 2013) . Recent studies have demonstrated HBX inhibition of the expression of miRNA-15b, which otherwise directly targets the fucosyltransferase 2 enzyme and increases the levels of the tumor-associated antigen Globo-H, ultimately enhancing HCC cell proliferation (Wu et al., 2014) . HBX has also been shown to decrease the inhibitory effect of miRNA-205 on carcinogenesis by down-regulating the expression of miRNA-205 in the livers of HBX transgenic mice (Zhang et al., 2013) .

Although several reports have provided evidence for a relationship between HBX, specific miRNAs, and target genes (Esteller, 2011; Xu et al., 2013; Zhang et al., 2013; Wu et al., 2014) , the regulation of the global miRNA profile in liver cells by HBX in relation to the development and progression of HCC remains to be fully clarified. Therefore, this study was designed to use microarray analysis to investigate the alteration of miRNA profiles in L02 and HepG2 cell lines that were transfected with HBXexpressing lentivirus, and to compare the results to those from control L02 and HepG2 cells transfected with empty lentivirus. The resulting set of differentially expressed miRNAs were subject to target gene prediction and pathway analysis. Finally, the internetwork of the HBXinduced miRNAs pathway was investigated to determine whether HBX-induced mRNAs function in a synergistic manner to support the pathogenesis of HCC.

## 2. Materials and methods

### 2.1. Cell lines

L02, HepG2, and HepG2.2.15, which was stably transfected with 2.1-fold HBV genome DNA, were permissible to HBV proliferation in cells (Sells et al., 1987) (all from the Chinese Academy of Sciences). The cells were cultured in a complete growth medium supplemented with 10% fetal bovine serum in a humidified atmosphere with 5% CO 2 and a temperature of 37 °C.

### 2.2. Tissues

All the patients underwent surgical resection of primary HCC at the Institute of Hepatobiliary Surgery, Southwest Hospital, Army Medical University (Third Military Medical University). The HCC tissues and the adjacent tissues were diagnosed by pathological identification of the Department of Pathology, Southwest Hospital. Patient-derived HCC tissues were obtained from patient tumor specimens with informed consent according to the protocols approved by the Institutional Review Board of the Southwest Hospital, Army Medical University (Third Military Medical University) (Chongqing, China).

### 2.3. Lentivirus-mediated transfection of HBX

The cells were seeded into 24-well plates at a density of 1 × 10^4^ cells/well. After 1 day of culture, the indicated combinations and multiplicity of infection (MOI) ratios of control (empty vector) or recombinant lentiviruses expressing HBX and GFP were applied to the cells according to the procedure reported by Chiang et al. (2014) . The virus-infected L02 and HepG2 cells were then cultured in fresh 24-well plates for the indicated times, with medium exchange every other day.

### 2.4. Total RNA extraction, qRT-PCR, and microarray analyses

Total RNA was extracted from the cells and clinical tissues (from Southwest Hospital, China) by using the RNAiso reagent (TaKaRa, Japan). The miRNA microarray was designed and detected by the Kangchen Company (China). To validate the microarray findings, cDNA synthesis was performed using a PrimeScript RT Reagent Kit (TaKaRa) and the products were amplified by qPCR with SYBR Premix Ex Taq II (TaKaRa), the appropriate miRNA primers (TianGen Company, Beijing, China) and the Stratagene Mx3000P real-time PCR system (Agilent Technologies, CA, USA). The qPCR-detected expression levels were normalized to those of the snRNA U6 endogenous control using the 2−∆Ct method.

### 2.5. Immunoflourescent and immunohistochemical staining

For immunoflourescent staining, the cells were simultaneously incubated with the primary rabbit antimouse hepatitis B virus X antigen antibody (Abcam, UK) and the secondary antirabbit IgG antibody (Abcam). For immunohistochemical staining, endogenous peroxidase activity was quenched by incubating the slide with 0.6% hydrogen peroxide, after which the slides were washed in phosphate-buefred saline and exposed to the primary rabbit antimouse hepatitis B virus X antigen antibody (Abcam). After the incubation, the slides were washed and exposed to the secondary Alexa Fluor® 555 conjugated antirabbit IgG (Abcam). Then, the slides were washed and exposed to the avidin–biotin complex for 30 min at room temperature. Also, the immunoreactions were detected by use of the diaminiobenzidene reagent (Sigma, China).

### 2.6. Target gene prediction

Microcosm (http://www.ebi.ac.uk/enright-srv/ microcosm/), miRanda (http://microRNA.org), and Targetscan (http://www.targetscan.org) programs, which apply computational algorithms based on base-pairing rules for miRNA binding to mRNA target sites, the location of binding sequences within the 3’-UTR of the target, and the conservation of target binding sequences within the related genomes, respectively, were used to predict target genes for the differentially expressed miRNAs. The genes identified by these software programs were taken as target genes. The predicted genes were subjected to pathway analysis (http://www.kegg.jp/) using the standard enrichment computation method.

### 2.7. Gene pathway analysis

The functional analysis of the predicted genes was carried out by mapping to KEGG pathways (http://www.kegg.jp/). The EASE-score, the Fisher’s P-value, or the hypergeometric P-value was calculated to determine the significance of the pathway’s correlation with the conditions. The lower the P-value, the more significant the pathway’s correlation; a P-value cut-of of 0.05 was used.

### 2.8. miRNA-pathway analysis

Although 19 significantly differentially expressed miRNAs were found, only when the overlap coefficient was ≥0.5 and the overlap numbers of the pathways of miRNAtargeted gene was >3, were the eligible miRNAs selected for inclusion in the analysis of the miRNA-pathway network. This strategy of miRNA selection was represented by the following equation: given the sets X and Y, and the cardinality operator ||where|X|equals the number of elements within the set X, the overlap coefficient was defined as overlap (X,Y)=|X∩Y|/min(|X|,|Y|) (Merico et al., 2010) . X remains to one collection of the target gene, while Y remains to another collection of the target gene. The miRNA-pathway network analysis was performed by the CytoScape software.

### 2.9. Statistical analysis

Data generated from the microarray was imported to Microsoft Excel. After normalizing the signal of each miRNA by using global average normalization as described by Bilban et al. (2002) , the expression level of each miRNA was calculated. Student’s t-test was performed to estimate between-group differences. All the statistical analyses were performed using the GraphPad Prism software 5.0. Statistical significance was defined by a P-value of <0.05. For each miRNA, the difference between HCC and normal liver cells was considered significant if the fold-change was >2 or <0.5 and the P-value was <0.05.

## 3. Results

### 3.1. HBX-induced aberrant miRNAs in normal liver cells and HCC cells

**Figure 1:**
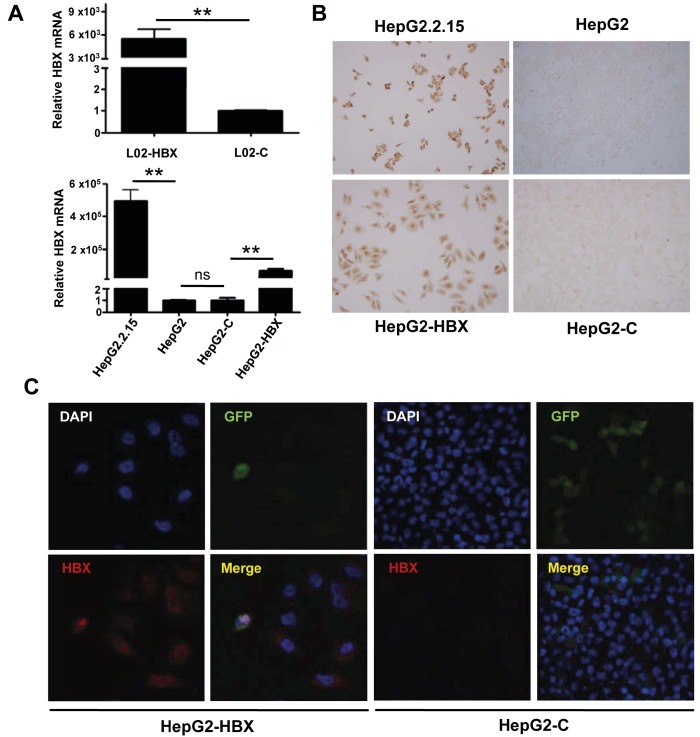
Establishment of HBX expressing HepG2 and L02 cell lines. A: HBX mRNA level in L02 and HepG2 cells transfected with empty lentivirus (C) or recombinant lentivirus (HBX) was detected by qRT-PCR. B: HBX protein expression was detected by immunohistochemical staining of untransfected HepG2.2.15 and HepG2 cells, HepG2-HBX cells and HepG2-C cells. C: Immunofluorescent analysis of HBX protein showed localization of the ectopically expressed HBX from the recombinant lentivirus and lack of HBX in the cells transfected with empty vector. *P < 0.05, **P < 0.001; ns, not significant.

To define the global profile of HBX-induced alterations in miRNA of liver cells in relation to HCC, we first established HBX-overexpression cell lines by infecting L02 and HepG2 cells with empty lentivirus vector or recombinant lentivirus, respectively. The qPCR analysis indicated that HBX mRNA was elevated thousands of times higher in the L02 cells transfected with HBX-expressing lentivirus, as compared with the L02 cells transfected with the empty lentivirus (Figure 1A). The qRT-PCR results were similar for the HepG2 cells, although the HBX mRNA expression level in the HepG2-HBX cells was lower than that in the HepG2.2.15 cells (Sells et al., 1987) (Figure 1A). The ectopic expression of the HBX protein, from the HBX gene encoded by the recombinant lentivirus vector, was verified in the transfected cells by immunohistochemical staining (Figure 1B). Immunoflourescent staining confirmed that the HBX protein was expressed in both nuclei and the cytoplasm, consistent with previous reports (Majano et al., 2001; Shirakata and Koike, 2003) . The HepG2 cells that were transfected with empty lentivirus (lacking the HBX gene, but encoding the GFP gene) did not express any HBX protein that was detectable by either immunohistochemistry or immunofluorescence, although the GFP protein was appropriately expressed (Figure 1C).

**Figure 2:**
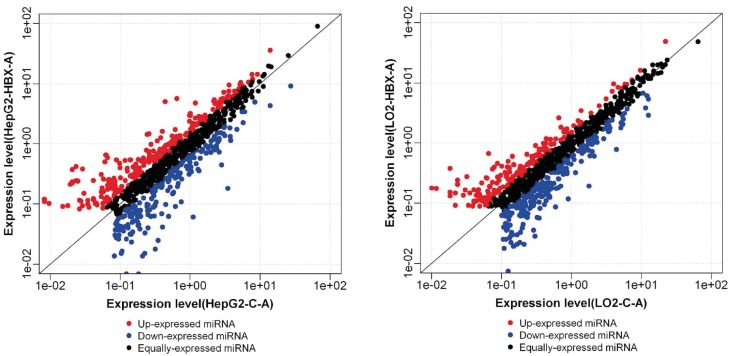
Differential expression of HBX-induced miRNAs in normal liver and HCC cells. Scatter plots of up- and downregulated miRNAs in control (x-axis) and HBX over-expressing (y-axis) cells. Each point in the figure represents a single miRNA. The red points represent upregulated miRNAs with a ratio of >2. The black points represent equally-expressed miRNAs with a ratio of ≥1/2 and ≤2. The blue points represent downregulated miRNAs with a ratio of <1/2.

Upon the microarray analysis of these four cell lines (L02-empty lentivirus, L02-recombinant lentivirus (L02HBX), HepG2-empty lentivirus, HepG2-recombinant lentivirus (HepG2-HBX)), miRNAs with ≥2-fold-change differential expression were selected as the candidate target miRNAs of HBX. The up- and downregulated candidate target miRNAs were presented in Figure 2, and were assessed to determine the key HBX-induced miRNAs that may play pivotal roles in both normal liver and HCC cells. The comparison of the differential miRNA profiles of the L02-HBX cells and the HepG2-HBX cells showed 15 miRNAs that were simultaneously decreased (2- to 36fold) and 4 miRNAs that were simultaneously increased (2.0- to 10.6-fold) in these cells (Table).

### 3.2. Validation of candidate miRNAs induced by HBX

**Figure 3:**
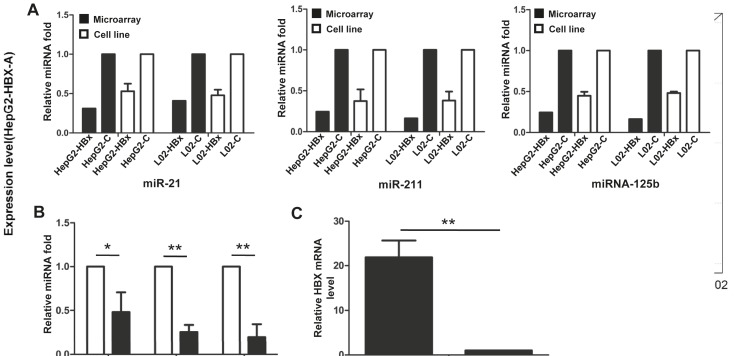
Validation of miRNA expression by qRT-PCR. A: The relative expression of miRNA-21, miRNA-211, and miRNA-125b in L02 and HepG2 cells transfected with empty lentivirus (C) or recombinant lentivirus (HBX). B: The relative expression level of miRNA-21, miRNA-211, and miRNA-125b in HBV-infected HCC samples (Ca) and the adjacent tissues (Aj). C: The relative HBX mRNA expression in HBV-infected HCC samples and the adjacent tissues. *P < 0.05; **P < 0.001.

In order to validate the microarray results, we selected three miRNAs for qPCR verification. The results showed that the expression levels of miRNA-21, miRNA-211, and miRNA-125b—among the 15 downregulated candidate miRNAs (Table)—were decreased about 0.5-, 0.46-, and 0.378-fold-change, respectively, as compared to the vectors alone. These results were consistent with the microarray results (Figure 3A). Furthermore, the expression levels of these three miRNAs in surgery specimens from 10 HCC patients with HBV infection were also significantly reduced compared to the levels detected in paired adjacent nontumor tissues (Figure 3B); in addition, the level of HBX mRNA was significantly higher in the tumor tissues than the adjacent nontumor tissues (Figure 3C). These in vitro and in vivo results indicated that miRNA-21, miRNA-211, and miRNA-125b were downregulated in the presence of HBX, thus supporting the accuracy of the data generated from the microarray (Table).

**Table:** 

Name	Fold-change		Location
	HepG2-HBX vsHepG2-C	L02-HBX vsL02-C	
Upregulated			
hsa-miR-4436b	10.57	2.010521666	Chr2: 110086433-110086523
hsa-miR-5584	2.521	2.287049727	Chr1: 44545493-44545552
hsa-miR-663a	2.09	4.009075941	Chr20: 26208186-26208278
hsa-miR-4776	2.00	3.958031355	Chr2: 212926257-212926336
Downregulated			
hsa-miR-4796	0.496919918	0.473770783	Chr3: 114743445-114743525
hsa-miR-211	0.470588235	0.429258291	Chr15: 31065095-31065116
hsa-miR-25	0.439732143	0.059836954	Chr7: 100093560-100093643
hsa-miR-4489	0.428776978	0.027965903	Chr11: 65649192-65649253
hsa-miR-4447	0.411417323	0.432633765	Chr3: 116850277-116850367
hsa-miR-622	0.406779661	0.487823306	Chr13: 90231182-90231277
hsa-miR-4283	0.327731092	0.31295177	Chr7: 56955785-56955864
hsa-miR-132	0.322946176	0.359399797	Chr17: 2049908-2050008
hsa-miR-219-2	0.3125	0.373606984	Chr9: 128392618-128392714
hsa-miR-21	0.310725552	0.408140061	Chr17: 59841266-59841337
hsa-miR-4638	0.300433839	0.328614016	Chr5: 181222566-181222633
hsa-miR-1909	0.294372294	0.472577776	Chr19: 1816159-1816238
hsa-miR-125b-1	0.244444444	0.163997579	Chr11:122099757-122099844
hsa-miR-632	0.227272727	0.302395535	Chr17: 32350109-32350202
hsa-miR-26a-2	0.125348189	0.344113776	Chr12: 57824609-57824692

### 3.3. Candidate target genes of miRNAs induced by HBX

**Figure 4:**
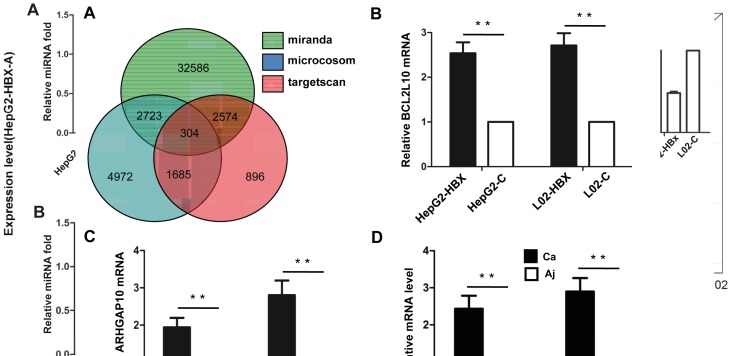
Prediction and validation of target genes of HBX-induced miRNAs. A: Summary of the predicted miRNA target genes identified by three algorithms: TargetScan, miRanda, and Microcosom. The numbers in the Venn diagram indicate the target genes predicted by each algorithm and the overlap among them. B, C: The relative mRNA expression of BCL2L10 and ARHGAP10 genes, respectively, in L02 and HepG2 cells transfected with empty lentivirus (C) or recombinant lentivirus (HBX) determined by qRT-PCR. D: The relative mRNA level of HBX in HBV-infected HCC samples (Ca) and the paired adjacent tissues (Aj) determined by qRT-PCR. *P < 0.05; **P < 0.001.

Prediction analysis by three different computational algorithms identified 38,187, 9684, and 5459 target genes, respectively, as targets of the total set of HBX-altered miRNAs. A total of 304 target genes overlapped among these three algorithms, as shown by the Venn diagram (Figure 4). The overlapping target genes included some important genes that have been previously verified by other studies in the literature, including B-cell lyphoma-3 (BCL3) (Ahlqvist et al., 2013) and cytidine deaminase (CDA) (Zauri et al., 2015) . The overlapping target genes also included many genes that have yet to be verified; therefore, we selected two candidate target genes— BCL2L10 and ARHGAP10—to verify their expression in vitro and in vivo.

BCL2L10 was identified as a potential target of miRNA125b, and ARHGAP10 was identified as a potential target of miRNA-21 and miRNA-211. According to previous reports, BCL2L10 can induce cell apoptosis through a mitochondrial signaling pathway under conditions of gastric cancer (Xu et al., 2011) and ARHGAP10 can contribute to the adherens junction (Sousa et al., 2005) . By using the qRT-PCR assay, we found that the marked decrease of the three miRNAs cited above as observed in HepG2-HBX and L02-HBX cell lines (Figure 3) was accompanied by significant up-regulation of both BCL2L10 and ARHGAP10 (Figure 4B and 4C). In vivo analysis of liver tissues confirmed this finding, with the mRNA expression level of BCL2L10 and ARHGAP10 being shown as significantly increased in HBV-infected liver tissues (Figure and 4D).

### 3.4 Analysis of HBX-induced miRNA target genes according to KEGG pathways

**Figure 5:**
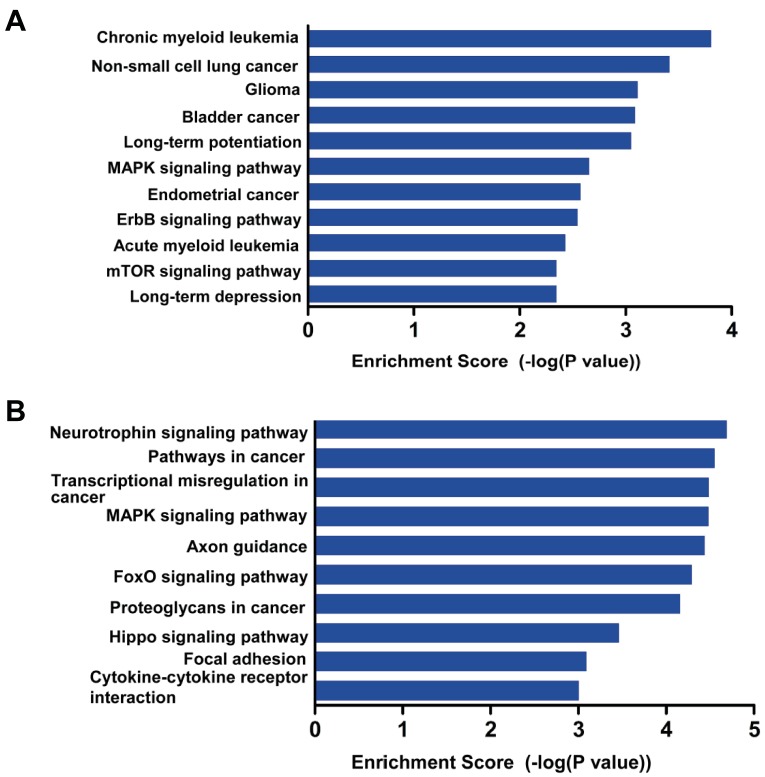
KEGG pathway analysis of target genes of the HBX-induced differentially expressed miRNAs. A, B: The top 10 enriched pathways for the upregulated and downregulated miRNAs, respectively.

Because signaling pathways play key roles in many biological and pathological events, we performed enrichment analysis of the KEGG pathways for each of the HBX-induced miRNA target genes. The top 10 enriched KEGG pathways for the genes related to the upregulated miRNAs were involved in chronic myeloid leukemia, nonsmall cell lung cancer, glioma, bladder cancer, long-term potentiation, the MAPK signaling pathway, endometrial cancer, the ErbB signaling pathway, acute myeloid leukemia, and the mTOR signaling pathway (Figure 5A). For the genes related to the downregulated miRNAs, the top 10 enriched KEGG pathways for the target genes were involved in the neurotrophin signaling pathway, pathways in cancer, transcriptional misregulation in cancer, the MAPK signaling pathway, axon guidance, the FoxO signaling pathway, proteoglycans in cancer, the Hippo signaling pathway, focal adhesion, and cytokine-cytokine receptor interaction (Figure 5B). Among these enrichment pathways, most were cancerrelated (e.g., acute/chronic myeloid leukemia, bladder cancer, endometrial cancer, and proteoglycans in cancer), suggesting that these pathways might represent potential host mechanisms that were manipulated by the virusencoded HBX through its regulation of host miRNAs and leading to HCC.

### 3.5 Pathways-based miRNAs internetwork induced by HBX

**Figure 6:**
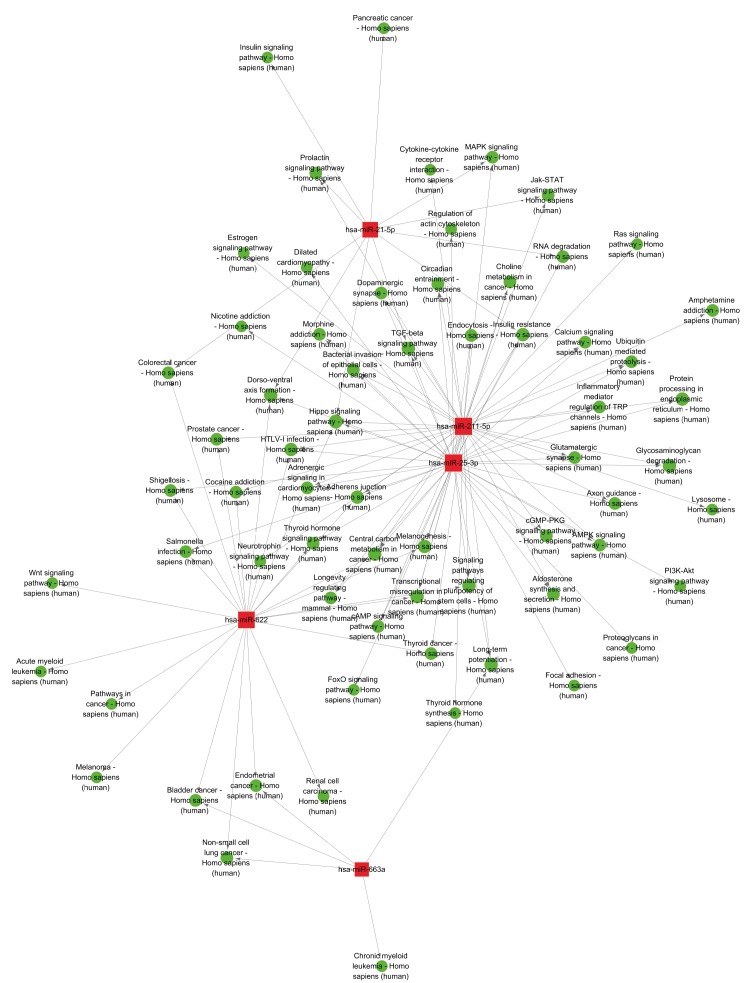
HBX-induced miRNAs internetwork analysis. The red square nodes represent the eligible miRNAs; the green circular nodes represent the specific pathways (function terms). The arrows indicate the direction of relationships.

In order to directly show the roles of differentially expressed miRNAs on pathways, we generated a network between the miRNAs and potential pathways by using the CytoScape software. The miRNAs were selected only when the overlap-coefficient was ≥0.5 and the overlap numbers of the pathways of miRNA-targeted gene were ≥3; the eligible miRNAs were thus included to make up the pathways-based miRNAs internetwork. A total of 5 miRNAs were identified as interacting with others and found to mediate at least 3 common pathways (Figure 6). Among these, only miRNA-663a, one of the total four upregulated miRNAs fitting the criteria for this analysis (overlap-coefficient of ≥0.5 and overlap numbers of the pathways of miRNA-targeted gene of ≥3), formed an internetwork with the other miRNAs. In addition, among the 15 downregulated miRNAs that fit the criteria for this analysis, only miRNA-21, miRNA-211, miRNA-25, and miRNA-622 were involved in the network (Figure 6).

## 4. Discussion

In the present study, we performed microarray to investigate the HBX-induced differential expression pattern of miRNAs in human liver cells in relation to HCC. We identified a total of 19 miRNAs that showed significant changes in expression following ectopic HBX induction. Five of the 19 miRNAs (miRNA-132, miRNA-219, miRNA-125b, miRNA-26a, and miRNA-21) had been previously demonstrated to be associated with HCC (Wei et al., 2013; Zhou et al., 2014; Song et al., 2015; Zhou et al., 2015) . Based on the KEGG pathway analysis, we found that the target genes of these miRNAs were closely associated with tumor-related biological processes (Figure 5). We also demonstrated in this study that HBX might exert pathological effects through miRNAs that target common pathways in hepatic cells (Figure 5).

Some of the HBX-induced miRNAs and their target genes predicted in the current study were mapped for their putative cooperation with each other in liver cells. In particular, 5 of the miRNAs—miRNA-663a, miRNA-21, miRNA-211, miRNA-25, and miRNA-622—had the potential to regulate common pathways in conjunction with the others, which were involved in several cancerrelated pathways including colorectal cancer, gastric cancer, and HCC ( Guo et al., 2011; Fang et al., 2015; Song et al., 2015; Xu et al., 2015) (Figure 6).

Therefore, this study provides new potential markers of clinical diagnosis and/or therapeutic targets of HCC.

## Acknowledgments

This study was funded by grants from the General Program of NSFC (Nos. 31670889 and 31200668).
